# Material Translation Based on Neural Style Transfer with Ideal Style Image Retrieval

**DOI:** 10.3390/s22197317

**Published:** 2022-09-27

**Authors:** Gibran Benitez-Garcia, Hiroki Takahashi, Keiji Yanai

**Affiliations:** 1 Graduate School of Informatics and Engineering, The University of Electro-Communications, Chofugaoka 1-5-1, Chofu-shi 182-8585, Japan; 2 Artificial Intelligence eXploration Research Center, The University of Electro-Communications, Chofugaoka 1-5-1, Chofu-shi 182-8585, Japan

**Keywords:** material translation, neural style transfer, instance normalization, human perception of materials

## Abstract

The field of Neural Style Transfer (NST) has led to interesting applications that enable us to transform reality as human beings perceive it. Particularly, NST for material translation aims to transform the material of an object into that of a target material from a reference image. Since the target material (style) usually comes from a different object, the quality of the synthesized result totally depends on the reference image. In this paper, we propose a material translation method based on NST with automatic style image retrieval. The proposed CNN-feature-based image retrieval aims to find the ideal reference image that best translates the material of an object. An ideal reference image must share semantic information with the original object while containing distinctive characteristics of the desired material (style). Thus, we refine the search by selecting the most-discriminative images from the target material, while focusing on object semantics by removing its style information. To translate materials to object regions, we combine a real-time material segmentation method with NST. In this way, the material of the retrieved style image is transferred to the segmented areas only. We evaluate our proposal with different state-of-the-art NST methods, including conventional and recently proposed approaches. Furthermore, with a human perceptual study applied to 100 participants, we demonstrate that synthesized images of stone, wood, and metal can be perceived as real and even chosen over legitimate photographs of such materials.

## 1. Introduction

Since the introduction of AlexNet [1] in the early 2010s, Convolutional Neural Networks (CNNs) have become the central pillar of computer vision. Over the next decade, the field gradually shifted from engineering features to designing CNN architectures. This success is attributed to more efficient graphics processing units (GPUs), new regularization techniques, and data augmentation methods to generate more training samples by deforming the available datasets. CNN architectures are now leading the performance of almost all computer vision tasks, such as detection, segmentation, and recognition of different types of objects and regions in images and videos [2,3]. On the other hand, in 2016, Gatys et al. [4] first studied how to use CNNs for applying painting styles to natural images. They demonstrated that is possible to exploit CNN feature activation to recombine the content of a given photo and the style of artwork. Specifically, a pre-trained CNN architecture is used to extract content and style features from each image. Subsequently, the resultant image is optimized by minimizing the features’ distance iteratively. This work opened up the field of Neural Style Transfer (NST), which is the process of rendering image content in different styles using CNNs [5].

**Figure 1:**
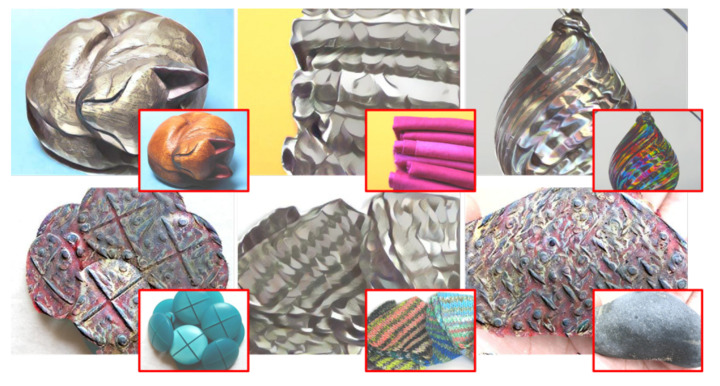
Examples of material translation results. Metal objects translated from different original materials: wood, fabric, glass, plastic, fabric, and stone (content image in red). From left to right and top to bottom, respectively.

NST has led to interesting applications that enable transforming the reality that human beings perceive, such as photo editing, image colorization, makeup transfer, material translation, and more [6,7,8,9]. In particular, material translation aims to transform the material of an object (from a real photograph) into a target material synthesized from the reference image (from now on, just called the style image). Consequently, the generated images can change the human perception of the objects, as shown in Figure 1. In these examples, objects made from light materials such as fabric or wood can be perceived as heavy materials, such as metal. Further, this technique can be combined with Augmented Reality (AR) and Virtual Reality (VR) devices to develop applications that generate alternate reality experiences.

**Figure 2:**
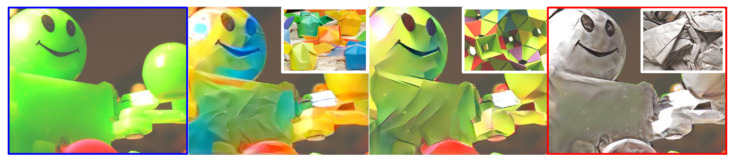
Examples of material translation (plastic → paper) using different style images (right corner of each generated result). The first picture shows the content image (blue), and the last is the generated image using our proposed framework (red).

An important issue of material translation is that the quality of the synthesized results totally depends on the chosen style image. For example, Figure 2 shows the results of material translation from plastic to paper using three different style images. From this figure, we can see that although the style images clearly show characteristics of paper, not all translation results can be recognized as paper toys. It is worth noting that the translation has to be localized only in the object region, keeping the background unaltered. Therefore, both problems need to be tackled to achieve realistic results that can challenge the perception of original objects.

In general, we can summarize three crucial aspects to achieve realistic results for material translation. The chosen style image must be able to (i) represent the target material clearly and (ii) share semantic information with the original object (similarity with the content image). Moreover, (iii) the original object must be segmented to maintain the background. Taking into account these aspects, in this paper we propose a material translation method based on NST and automatic style image retrieval. In this way, we cover the problems related to style image selection, i.e., (i) and (ii). Furthermore, we apply real-time material segmentation as a postprocessing step to fulfill (iii).

Our material translation method is defined as follows. In order to select an *ideal style image*, we firstly refine the search process by automatically choosing the most discriminative candidate images from each material class available. Secondly, we propose to remove the style information using instance normalization whitening (IN [10]) from the query (content) and the refined images (style) of the desired material. Thus, the final search is performed using normalized CNN features extracted from the VGG19 network [11]. Finally, to translate materials to object regions, we combine semantic segmentation with NST. Specifically, we obtain pseudo labels with a weakly supervised segmentation (WSS) framework [12] to train a real-time material segmentation model [13]. Thus, we can efficiently segment target regions (objects) to translate the material of the retrieved style image.

**Figure 3:**
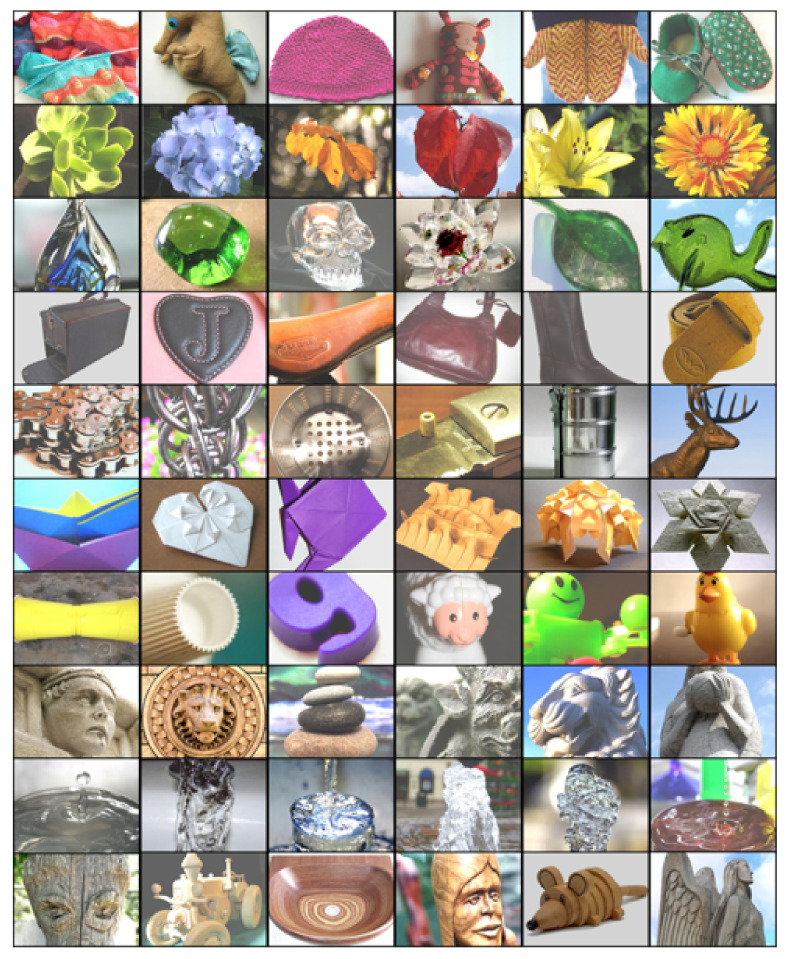
Example images from the ten material classes used in this paper. Each row depicts images from the same class, from top to bottom: fabric, foliage, glass, leather, metal, paper, plastic, stone, water, and wood.

In this paper, we employ ten different material classes shared in two publicly available datasets: Flickr Material Database (FMD [14]) and the Extended-FMD (EFMD [15]). Some examples from these datasets are shown in Figure 3. We quantitatively evaluate our work on different metrics, including: Inception Score (IS), Frechet Inception Distance (FID), classification accuracy, and segmentation performance. Qualitatively, we show examples of synthesized images that can be evaluated by visual inspection. Furthermore, we conduct a human perceptual study to evaluate the realism of the generated results. The study is designed to analyze the capacity to fool human perception by translating the original materials of target objects. One hundred participants strictly evaluated image triplets from the same material, where two were real photographs, and one was translated from a different original material. Participants are asked to choose the image that they think does not belong to the mentioned material (strict question). Thus, if they do not pick the synthesized image, it means that the translated results are real enough to fool human perception. The results of our study indicate that using our NST-based approach, it is possible to generate images that can be recognized as real even over legitimate photographs, especially for objects made of stone, wood, or metal.

In our previous work [16], we tested an image retrieval method for improving material translation and found that NST is better than GAN-based generation models for material translation. Therefore, in this paper, we focus the analysis on the cutting-edge NST methods, including conventional [4,17,18,19] and recent approaches [20,21,22].

In summary, in this work, we extend our workshop paper [16] findings. Hence, the novel contributions of this paper are threefold:

- We propose a single-material translation framework based on real-time material segmentation and neural style transfer with automatic style image retrieval.
- We evaluate our proposed method with state-of-the-art (SOTA) NST methods, including Gatys [4], Johnson’s [17], AdaIN [18], WCT [19], LST [20], MetaStyle [21], and STROTSS [22].
- We present a human perceptual study applied to 100 participants to evaluate the capacity of our generated results to fool the human perception of objects with translated materials.

## 2. Related Work

Neural Style Transfer methods can be divided in two groups: image-optimization-based and model-optimization-based [5]. The seminal work of Gatys et al. [4] is part of the first group, since the style transfer is built upon an iterative image optimization in the pixel space. Specifically, the content is defined by features extracted from multiple layers of pre-trained CNN, and the style is by terms of the Gram matrix of features extracted from another set of layers. Recently, Style Transfer by Relaxed Optimal Transport and Self-Similarity (STROTSS [22]) was proposed as an alternative to Gatys team’s work. In this approach, the style is defined as a distribution over features extracted by CNN, and the distance is measured between these using an approximation of the earth mover’s distance. Further, the content is defined by using local self-similarity descriptors. With these original representations of content and style, STROTSS overcame the results of Gatys, which was for a long time considered the gold standard due to its visual quality [5].

To enable faster stylization, the second group of works trains Conv–Deconv Networks using content and style loss functions to approximate the results in a single forward pass. This method was first introduced by Johnson et al. with the well-known perceptual loss function [17]. An important drawback of Johnson’s approach is that an independent model must be trained for each single style image. Therefore, some approaches aim to train one single model to transfer arbitrary styles [18,19,20]. Huang and Belongie [18] propose adaptive instance normalization (AdaIN) to achieve real-time performance. AdaIN transfers channel-wise statics between content and style, which are modulated with affine parameters (trainable). Concurrently, Li et al. [19] propose a pair of whitening and coloring transformations (WCT) to achieve the first style learning-free method. In the same line, Linear Style Transfer (LST) [20] is proposed as an arbitrary style transfer that learns the transformation matrix with a feed-forward network and presents general solutions to the linear transformation approaches (such as AdaIN and WCT). It is known that usually arbitrary style transfer models come at the cost of compromised style transfer quality compared to single-style model methods [5]. To overcome this issue, the recent MetaStyle approach [21] formulates NST as a bi-level optimization problem, which is solvable by meta-learning methods. MetaSttyle combines arbitrary style representation learning with only a few post-processing update steps to adapt to a fast approximation model with quality comparable to that of image-optimization-based methods. A more recent approach called IFFMStyle [23] introduced invalid feature filtering modules (IFFM) to an encoder–decoder architecture for filtering the content-independent features in the original and generated images. In this way, IFFMStyle is able to transfer the style of a collection of images rather than selecting a single style image. On the other hand, Total Style Transfer [24] resolves the limitation of transferring the scale across style patterns of a style image by utilizing intra/inter-scale statistics of multi-scaled feature maps. The process is achieved by a single decoder network using skip-connections to efficiently generate stylized images. It is worth noting that all mentioned methods from both groups can be applied to material translation. Hence, we test our framework with different SOTA NST methods to find the most suitable approach for our task.

## 3. Proposed Method

**Figure 4:**
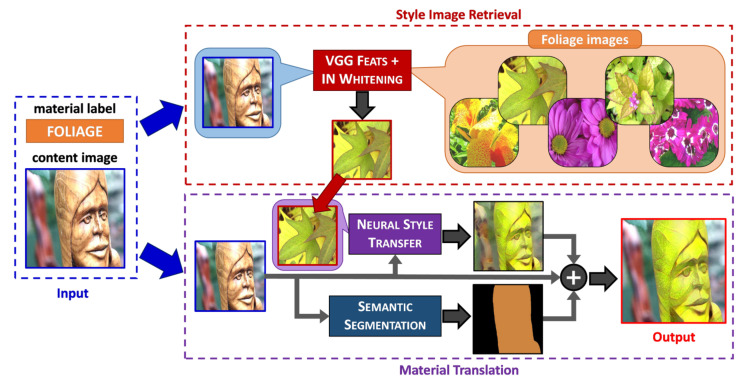
General overview of our proposal for material translation using style image retrieval.

Matsuo et al. [9] proposed combining conventional NST [4] with a weakly semantic segmentation (WSS) approach [25] to achieve realistic material translation results. Therefore, we build upon Matsuo’s framework and extend it as follows: (1) we propose automatic image retrieval rather than manually finding the *ideal style image*; (2) we employ a real-time semantic segmentation model trained with pseudo labels generated with a SOTA WSS method; and (3) we analyze different SOTA NST approaches since we previously found that NST-based strategies usually generate more realistic results than the GAN-based methods [16]. Figure 4 illustrates our proposed inference process for material translation focused on a single object (wood → foliage). As an input, we take the content image and the label of the target material. Our main contribution resides in the style image retrieval process, where we propose to apply IN whitening to remove the style information and retrieve the *ideal style image* based on its semantic similarity with the content image. Subsequently, in the material translation stage, we apply the NST approach to synthesize the material of the content image using the retrieved style. At the same time, we apply semantic segmentation on the content image to get the foreground mask depicting the material region that will be translated. Finally, the output is generated by combining synthesized and content images using the foreground mask. In the following subsections, we describe both of the main stages: Style Image Retrieval and Material Translation.

### 3.1. Style Image Retrieval

**Figure 5:**
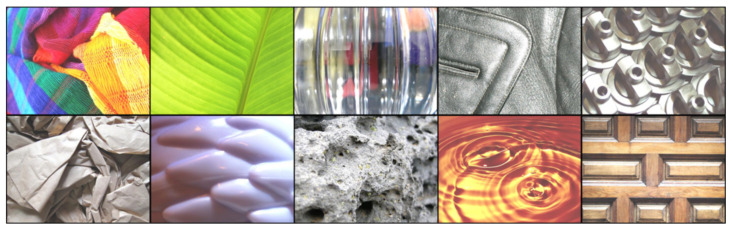
Fixed style images per material selected from the best-scored samples and the widest material regions. From left to right and top to bottom: fabric, foliage, glass, leather, metal, paper, plastic, stone, water, and wood.

We build our image retrieval process upon two key ideas: search refinement and style removal from CNN features. For search refinement, we assume that the *ideal style image* must reflect essential characteristics from its material while showing apparent differences from others. Therefore, to refine the style image search to the most discriminative samples, we train a CNN model to classify all possible style images from the target material to automatically choose the samples with the highest score (defined by a classification threshold $Th_{clf}$). Since this classification model is crucial to define high-quality candidates, we choose the robust CNN architecture of InceptionV3 [26]. Subsequently, we automatically choose the possible style images that present the widest area of the target material. To do so, we define the relative material area as the division of the material region by the image size, and we choose the samples with the most extensive regions using an area threshold $Th_{area}$. Note that the material region is depicted by the provided pixel annotation of the dataset or is automatically detected by a semantic segmentation model. Then, the style image search is refined to the best-scored images with more extensive material regions from the target material. In practice, we set $Th_{clf}$ and $Th_{area}$ both to 0.99, so that the number of refined images drops to about 16% of samples per material. Figure 5 shows some examples of possible ideal style images that satisfy our designed requirements.

Equally important, we employ instance normalization (IN) whitening for style removal, which was originally proposed to remove instance-specific contrast information from input images [10]. Huang et al. [27] experimentally proved that the distance between VGG [11] features of two samples is more domain-invariant when using IN whitening (experiment details on the supplementary material of [27]). In other words, the features of two images with the same content and different styles (domain) are closer in the euclidean space than those from the same style but with different contents. That is what we seek in our style image retrieval process: *to find the most similar style image based on its content (semantic) by excluding its style information.* Therefore, we build the style-free image retrieval on a VGG19, replacing all batch normalization (BN) layers with non-parametric IN. The formal definition of the IN is as follows:

(1) $$\begin{array}{c} y_{tijk}=\frac{x_{tijk}-\mu_{ti}}{\sqrt{\sigma_{ti}^{2}+\epsilon }}, \end{array}$$

where $x\in R^{T\times C\times W\times H}$ is an input tensor; $x_{tijk}$ denotes the tijk-th element, where *k* and *j* span spatial dimensions, *i* corresponds to the feature map (output from the current convolutional layer), and *t* is the index of the image in the batch; ϵ is an arbitrarily small constant used for numerical stability, and $\mu_{ti}$ and $\sigma_{ti}^{2}$, respectively are the per-instance mean and standard deviation, given by:

(2) $$\begin{array}{c} \mu_{ti}=\frac{1}{HW}\sum_{l=1}^{W}\sum_{m=1}^{H}x_{tilm},\\ \sigma_{ti}^{2}=\frac{1}{HW}\sum_{l=1}^{W}\sum_{m=1}^{H}{\left(x_{tilm}-\mu_{ti}\right)}^{2}, \end{array}$$

where *H* and *W* represent the height and width of the feature map, respectively. It is worth noting that, different from the conventional IN layer, we exclude the affine parameters. That’s why we call this process “whitening”.

**Figure 6:**
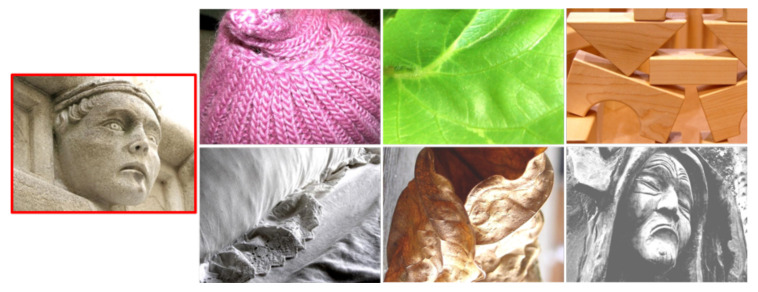
Retrieved results from our proposal using IN (**top**) and BN (**bottom**). From left to right: content image (stone); results of fabric, foliage, and wood materials.

We L2-normalize the VGG-features from the fc7 layer before using the euclidean distance to evaluate the similarity between the content (query) and the possible style image. Finally, the image with the lowest distance is retrieved (*ideal style image*). Note that we search only within the refined images from the target material, making the retrieval process very efficient. Figure 6 shows examples of the retrieved images from different materials by using IN or BN. As can be seen, the IN version retrieves style-free images that can be useful for material translation. Meanwhile, BN retrieves images that show apparent similarities to the content image (including color and style).

### 3.2. Material Translation with NST

In order to design a robust and efficient material segmentation model, we first obtain pseudo labels of object regions with a WSS approach; then, we train a real-time fully supervised semantic segmentation. Subsequently, material translation is achieved in three steps: (1) material translation with NST using the *ideal style image*; (2) real-time semantic segmentation of the content image; and (3) style synthesis to the segmented regions. Each sub-process is described below.

#### 3.2.1. Real-Time Material Segmentation

Since pixel annotation labels (semantic labels) are costly to acquire, WSS directly attacks the problem by generating segmentation labels of images given their image-level class labels. Particularly, Ahn and Kwak [12] propose to learn Pixel-level Semantic Affinity (PSA) from class activation maps (CAMs) [28] of a multi-label CNN network. The so-called AffinityNet predicts semantic affinity between a pair of adjacent image coordinates, and semantic propagation is done by a random walk. The training of AffinityNet is only supervised by the initial discriminative part segmentation (using CAMs), which is incomplete as a segmentation annotation but sufficient for learning semantic affinities within small image areas. Hence, we train AffinityNet with the Extended-FMD dataset, which contains image-level class labels only. As a result, we obtain coarse material region labels from the complete dataset (10,000 images), enough to train and fine-tune a case-specific semantic segmentation model.

On the other hand, Harmonic Densely Connected Network (HarDNet) [13] deals with real-time performance, an important issue of semantic segmentation methods. HarDNet achieves SOTA results by using harmonic densely connected blocks (HarDBlocks) instead of traditional bottleneck blocks [13]. A HarDBlock reduces most of the layer connections from a dense block, which heavily decreases concatenation cost. Moreover, the input/output channel ratio is balanced by increasing the channel width of a layer according to its connections. In particular, HarDNet for semantic segmentation is a U-shaped architecture with five encoder and four decoder blocks built of HarDBlocks. Compared to SOTA CNN architectures, HardDNet achieves comparable accuracy with significantly lower GPU runtime. Therefore, our material segmentation model is based on a HarDNet architecture. Particularly, we train a HarDNet model with the coarse labels obtained by AffinityNet. Subsequently, we fine-tune the model with the FMD dataset. Note that fine-tuning helps to enrich the quality of the HarDNet segmentation results by employing (in the supervision) the pixel-level annotations provided by the FMD dataset.

#### 3.2.2. Material Translation

As a baseline, we use the conventional NST method from Gatys [4] for material translation, which uses a pre-trained VGG19 network to extract content and style features. The translated image is optimized by minimizing the features distance and their Gram matrices (correlation operations). Gatys et al. [4] experimentally proved that the Gram matrix of CNN activations from different layers efficiently represents the style of an image. As shown in Figure 4, we first translate the whole content image to the retrieved style. Finally, we integrate the material region of the synthesized image and the background region of the content image into the final output ($I_{out}$), which is defined by:

(3) $$I_{out}=I_{gen}I_{mask}+I_{org}\left(1-I_{mask}\right)$$

where $I_{gen}$ is the synthesized image, $I_{mask}\in \left\{0,1\right\}$ is the region mask obtained by HarDNet, and $I_{org}$ is the content image with the original object.

## 4. Experimental Results

### 4.1. Implementation Details

We use PyTorch 1.2 with CUDA 10.2 for all experiments. For all trained methods, we used their respective pre-trained models on ImageNet [29]. AffinityNet (PSA) employed Adam as the optimization method. On the other hand, for HarDNet and InceptionV3, we used Stochastic Gradient Descent (SGD) with weight-decay $5\times 10^{-4}$ and momentum 0.9 as the optimizer. The rest of the parameters and data augmentation techniques were chosen as described in their original papers: PSA [12], HarDNet [13], and InceptionV3 [26]. The input image size for each network was 448×448, 512×512, and 299×299 for PSA, HarDNet, and InceptionV3, respectively. Note that all methods were tested with images in their original resolution (512×384 for FMD and EFMD datasets). Finally, we measured the inference time of each method (excluding I/O time) on an Intel Core i7-9700K desktop with a single NVIDIA GTX 1080Ti GPU.

### 4.2. Datasets

In this paper, we use two publicly available datasets: Flickr Material Database (FMD) and the Extended-FMD (EFMD). FMD [14] consists of 10 materials (fabric, foliage, glass, leather, metal, paper, plastic, stone, water, and wood). Each class contains 100 real-world images. The samples were selected manually from Flickr and were manually annotated at pixel-level. Some examples of this dataset are shown in Figure 3. EFMD [15] contains the same materials but includes 1000 images per class (10,000 in total). The samples were picked as close as possible to the FMD images, and only image-level annotations are provided. Images from both datasets are real-world RGB photographs with a size of 512×384 pixels. As shown in Table 1, for each method, we used a different number of training and testing images from both datasets. As mentioned before, HarDNet is firstly trained with the complete EFMD (HarDNet-base) and then fine-tuned with the FMD dataset (HarDNet). Note that for the material translation experiment (NST-based methods), each of the 100 testing images (10 per class) is transformed into each material class. Hence, we evaluate the NST-based methods with 1000 synthesized images.

### 4.3. Ablation Study

We first evaluate our proposal with classification and segmentation metrics: average accuracy (acc) and mean Intersection over the Union (mIoU). The classification accuracy shows the percentage of synthesized images that can be correctly classified with the trained InceptionV3 model. The intuition behind this evaluation is that the higher the accuracy, the better the quality of synthesized images. On the other hand, the segmentation metric presents a similar evaluation focused on pixel-level accuracy. In other words, the mIoU metric is stricter since it measures the accuracy of the translated materials by region rather than taking a single decision from the whole image.

As a baseline, we select one fixed style image per material based on the best-scored images and the widest material regions. Figure 5 shows the selected style images from each class. Note that these ten images are used to translate the entire testing set. As a result, ten translated images are generated from each content sample; hence, we evaluate 1000 synthesized images in total (100 per class). On the other hand, we apply our style image retrieval only to the refined images (about 15 per class) of the target material. We evaluate the results of our proposal by replacing the IN whitening (VGG19-IN) with BN layers (VGG19-BN) and without the normalization process (VGG19). We also evaluate the results with (w/refine) and without search refinement (w/o refine), which means searching for the ideal style image within 90 images per class.

Table 2 presents quantitative results of all variations. We observe that IN whitening significantly improves the results compared to the vanilla VGG19 and the BN (11% of accuracy and 4% of mIoU). These results concur with our hypothesis that style information must be removed from VGG features to retrieve *ideal style images*. Further, search refinement plays an essential role in the retrieving process. It boosts the material translation performance of our VGG19-IN by more than 15%. Surprisingly, the fixed-style image performance is comparable to that of the retrieving-based approaches and even outperforms the BN and vanilla VGG19 variations. This issue suggests that there is still a place for improvement in the retrieving process (to find better style images).

**Figure 7:**
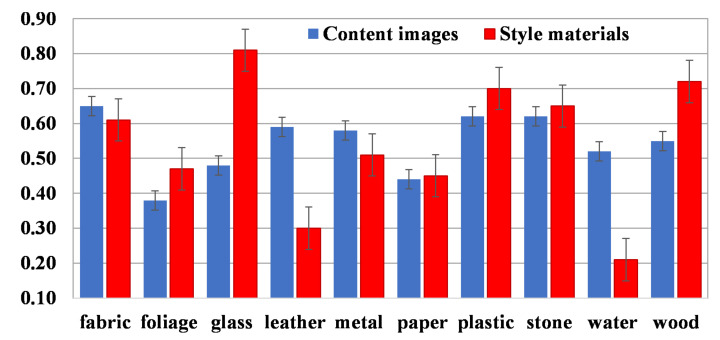
Classification accuracy per material class using our proposed VGG19-IN.

**Figure 8:**
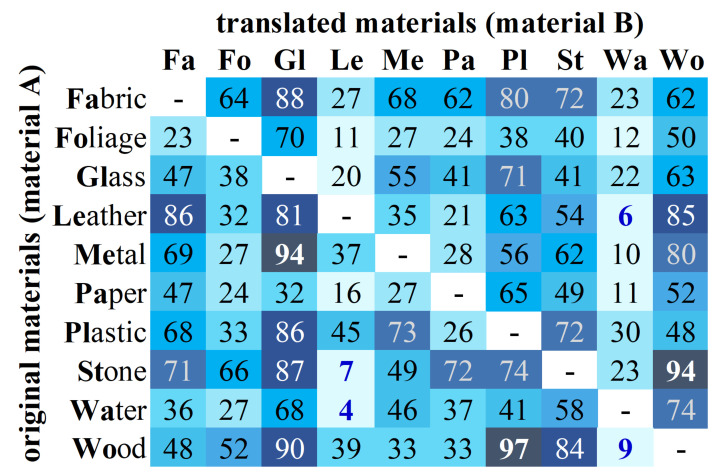
Classification accuracy (%) of translations from material A (rows) to material B (columns).

We also evaluate per-material performance from our proposal. Figure 7 shows the average accuracy of content (translated from original material to the ten classes) and style (individually translated material from all content styles) materials. As expected, not all materials show the same level of realism after the translation process. Interesting results are those from glass and water. The former seems to be easy to synthesize but challenging to translate, while the latter presents the opposite situation. Likewise, water and leather materials are challenging to synthesize, while glass and wood are certainly easier. Furthermore, in Figure 8, we evaluate the translation performance from each pair of materials (A → B), where rows and columns represent original (content) and translated (style) materials, respectively. Stone to leather and leather to water are challenging to translate, while stone to wood and wood to plastic are more accessible.

**Figure 9:**
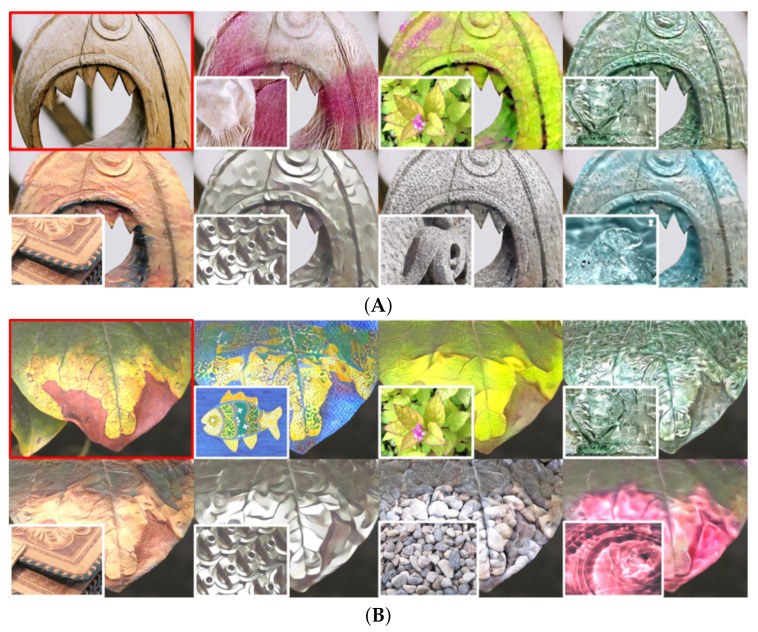
Translated results using our VGG19-IN proposal. (**A**) from wood, and (**B**) from foliage material (content image in red). From left to right, and top to bottom: content image, results of fabric, foliage, glass, leather, metal, stone, and water.

Figure 9 illustrates quantitative results of our VGG19-IN feature-based approach. We can see that all retrieved style images do not share style similarities with the content images explicitly (due to the IN whitening). Further, some of them show similar features: such as in the first example (from wood), the angular shape of the tooth-like part of the object has similar patterns on the foliage image. On the other hand, the difficulty in translating the water material might be related to the object shapes rather than the style itself. It is not natural to recognize water as certain shapes that do not exist in the real world.

### 4.4. Comparison among SOTA NST Methods

We evaluate our approach with the conventional methods of Gatys [4] and Johnson [17]; the real-time learning-free methods of AdaIN [18], WCT [19], and LST [20]; as well as the recently proposed methods of MetaStyle [21] and STROTSS [22]. In the case of the Johnson and MetaStyle models, using the default parameters provided in their respectively open-source codes, we train ten models based on the fixed style images shown in Figure 5. For Gatys and STROTSS, we optimize each content image with its respective *ideal styles* from all materials, generating the same number of images in total (100 per material). Finally, we use the respectively pre-trained models provided by the authors of AdaIN, WCT, and LST.

In addition to the acc and mIoU, we evaluated all methods using GAN metrics, i.e., Inception Score (IS), and the Frechet Inception Distance (FID). The IS estimates the quality of the synthesized images based on how well the InceptionV3 model classifies them. This metric combines the confidence of the conditional class predictions for each synthetic image (quality) and the integral of the marginal probability of the predicted classes (diversity). However, IS does not capture how synthetic images compare to real ones. That’s the main reason for introducing FID, which employs the coding layer of the InceptionV3 model to generate features from real and synthesized images. Thus, the distance between the distributions from both groups of images is then calculated using the Frechet distance. Finally, we use our pre-trained InceptionV3 model to calculate IS and FID metrics, and the final results are averaged over the 1000 synthesized images generated from the 100 content images. Note that as an accuracy score, the higher the IS is, the better. Contrarily, the smaller the FID is, the better, as it reflects the distance from real images.

**Figure 10:**
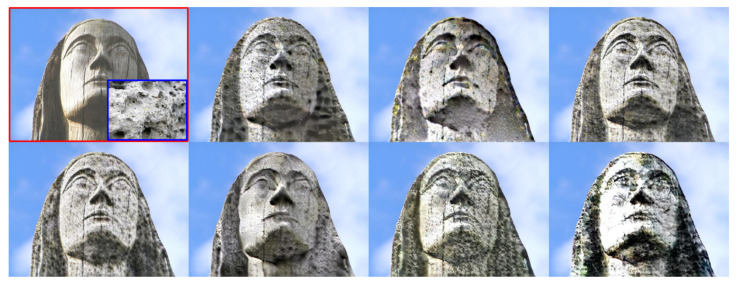
Qualitative results from all evaluated NST methods, translating from wood to stone. From left to right and top to bottom: content image (red) and style (blue); results from Gatys, STROTSS, Johnson, MetaStyle, WCT, LST, and AdaIN.

Table 3 shows the results from all evaluated NST methods. As expected, the best results are obtained by the image-optimization-based approaches (Gatys and STROTSS). However, due to their iterative optimization process, these are the methods with the slowest inference time. STROTSS obtained the best FID score, which means that the translated images share stronger semantic similarities with real photographs than those translated by the Gatys method. Still, the latter gets better classification and segmentation accuracy. On the other hand, Johnson’s, AdaIN, and MetaStyle are the most computationally efficient methods. Nevertheless, AdaIN can be preferred since it uses only a single model to transfer arbitrary styles. Finally, WCT and LST show similar performance, although LST is about two times faster than the former. Figure 10 shows qualitative results from all methods. We can see that in this example, almost all synthesized images show distinctive properties of the target material (stone), such as rough and porous texture rather than the polished surface of the original wood material. Even so, the results of Gatys and STROTSS look significantly more real than those of AdaIN and LST.

### 4.5. Human Perceptual Study

Well-designed perceptual experiments with human observers are the most reliable known methodology for evaluating the quality of synthesized images. Human perceptual studies for NST approaches usually analyze the results from different NST approaches [22,27,30,31]. However, they do not assess if the generated images can be perceived as real over legit photographs of the same category. Therefore, we design a human perceptual study to analyze the capacity of the synthesized images to fool human perception by translating the original materials with our NST-based proposal.

**Figure 11:**
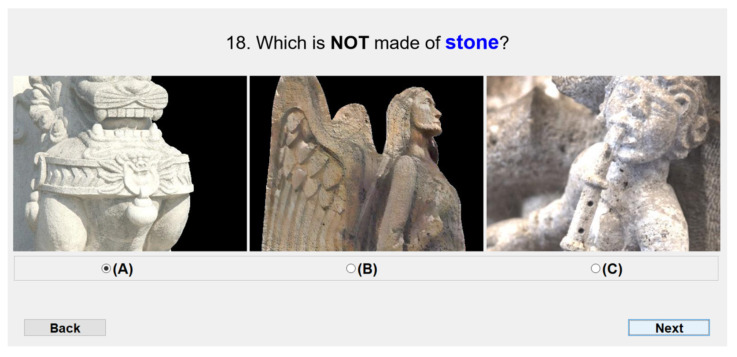
Human perceptual study interface: 70% of the participants chose (**A**), 18% chose (**C**), while the image generated using our approach (**B**) got only 12% of the votes.

Using the InceptionV3 model, for this study, we choose the top-6 synthesized images from each material, 60 images in total. An example of the top-6 translated results of metal are shown in Figure 1. These images are generated using the Gatys NST method and are usually translated from content images affine to the target material, as shown in the confusion matrix of Figure 8. We present each synthesized image along with two real photographs of objects from the same material. These photographs were manually selected from the FMD dataset and considered the objects included in the 60 synthesized images. Then, users were asked to select the image that does not belong to the depicted material from the three options. An example of the user interface is shown in Figure 11. Furthermore, to avoid biased results generated from the background of the original photographs, we remove this from all images used in the study. In this way, objects only found outdoors may have the chance to be recognized if these are translated to materials that are found indoors and vice-versa.

One hundred different participants took part in this study. We randomly showed 30 questions to each of them, keeping a ratio of 3 images per material. In total, 60 different questions were defined, and the image order of the triplets was also randomized to ensure fair comparisons. Each question was answered by 50 different participants, so we collected 3000 votes in total. Unlimited time was given to select the fake image out of three options. Note that there was not an option to indicate that all photos are real. Thus, the participants were forced to carefully find the outlier image. Consequently, if they did not pick the synthesized image, it means that the translated results are real enough to fool human perception.

**Figure 12:**
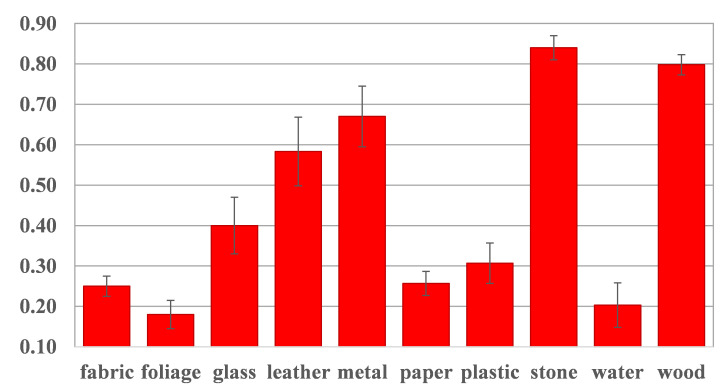
Realism results from the human perceptual study. *Y*-axis shows the average results when participants did not select the translated image as the outlier. Higher results represent more people being fooled by the synthesized images.

**Figure 13:**
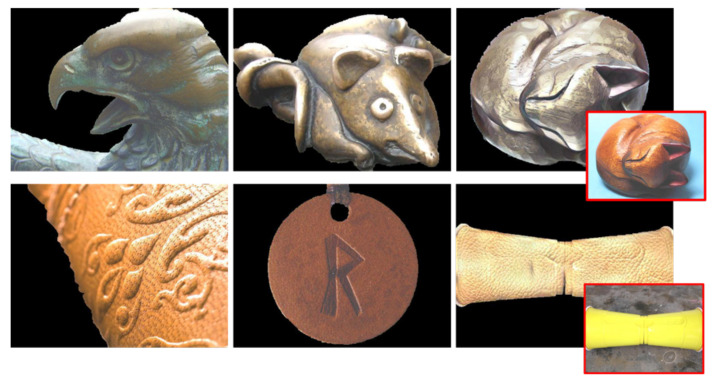
Examples of synthesized images with fewer votes (i.e., perceived as real). Each row shows the image triplets shown in one question (1st row: metal; 2nd row: leather). The most-voted pictures are shown from (**left**) to (**right**). The synthesized results of metal and leather got 4% and 14% of the votes, respectively (content image in red).

We counted the results when participants do not choose the synthesized images. Given that, the average results of the 3000 votes show that 44.86% of the time, participants took the translated results as representative pictures of their target material. These findings are more significant for some materials, as shown in Figure 12. Translated images from materials such as stone, wood, metal, and leather were taken as real over legitimate photographs by more than 50% of participants. In this way, we can prove that our NST-based approach can generate images that fool the human perception. Figure 13 shows some examples of the translated images that got the best acceptance in the human study. We can see that the synthesized images clearly exhibit elements from the target material, such as reflection and texture in the cases of metal and leather, respectively.

**Figure 14:**
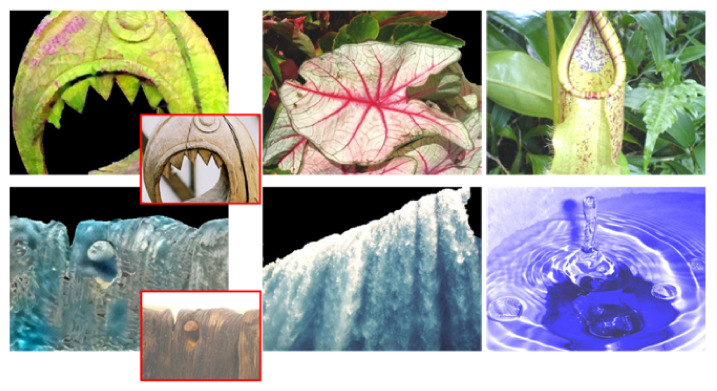
Examples of synthesized images with more votes (i.e., perceived as fake). Each row shows the image triplets shown in one question (1st row: foliage; 2nd row: water). The most-voted pictures are shown from (**left**) to (**right**). The synthesized results of foliage and water got 88% and 85% votes of the votes, respectively (content image in red).

On the other hand, although we selected the best-scored synthesized images, the results of foliage, water, and fabric were not able to fool the human perception. Figure 14 shows some examples of these materials. As we can see, the shape and texture of the original materials (wood in both cases) limits the results for being selected over legitimate photographs, even though the texture and color of the target materials are still present (foliage and water). Finally, we believe the results for plastic, paper, and glass can become more real if the original object shares similarities with authentic objects of the target material. However, with the current study, we cannot prove this hypothesis.

## 5. Conclusions and Future Work

In this paper, we introduced a material translation method based on real-time material segmentation and neural style transfer with automatic *ideal style image* retrieval. We build the image retrieval on VGG19 features whitened with instance normalization to remove the style information. Our results show that by excluding the style in the search process, the translated results are significantly better. We were able to translate the material of segmented objects using different NST methods, which we further analyzed quantitatively and qualitatively. Furthermore, we presented a human perceptual study to evaluate the quality of the synthesized images. The results of our study indicate that our NST-based approach can generate images of stone, wood, and metal that can be perceived as real even over legitimate photographs. Since we can alternate the material of some objects with the results being perceived as more real than fictional, we expect that our approach can be used to create alternate reality scenarios in which the user can feel a different environment based on the imperceptively modified objects.

As future work, we will further analyze different options to synthesize materials such as plastic, paper, and glass, which we believe can get more real if the original object shares similarities with authentic objects of the target material. Further, we would like to develop a real-time application that can translate the material of objects in-the-wild.

## Figures and Tables

**Table 1:** Number of training and testing images used for each method.

Method	Training Set	Test Set
PSA	10,000 (EFMD)	1000 (FMD)
HarDNet-base	10,000 (EFMD)	1000 (FMD)
InceptionV3	10,000 (EFMD)	1000 (FMD)
HarDNet	900 (FMD)	100 (FMD)
NST-based	-	100 (FMD)

**Table 2:** Classification and segmentation evaluation of the ablation study: “w/o” and “w/ refine” refers to without and with search refinement, respectively.

	w/o Refine		w/ Refine	
Method	acc	mIoU	acc	mIoU
Baseline	-	-	0.556	0.4860
VGG19-IN	**0.409**	**0.3967**	**0.572**	**0.5062**
VGG19-BN	0.291	0.3612	0.543	0.4887
VGG19	0.270	0.3520	0.506	0.4845

**Table 3:** Quantitative results of all evaluated NST methods. Inference time is measured on a single GTX 1080 Ti GPU.

Method	acc ↑	mIoU ↑	IS ↑	FID ↓	Inference Time ↓
Gatys [4]	**0.572**	**0.5062**	**4.181**	61.30	45.6545 s
STROTSS [22]	0.515	0.4887	4.046	**60.29**	89.1562 s
Johnson’s [17]	0.506	0.4464	3.887	68.44	**0.0881 s**
MetaStyle [21]	0.442	0.4674	3.635	61.93	0.1868 s
WCT [19]	0.353	0.4079	3.604	64.53	1.0151 s
LST [20]	0.343	0.3606	3.569	62.95	0.4816 s
AdaIN [18]	0.304	0.2780	3.129	74.52	0.1083 s

## Data Availability

Not applicable.
